# FEASIBILITY OF A SUPPORTIVE HOUSING PROGRAM FOR OLDER VETERANS WHO PREVIOUSLY EXPERIENCED HOMELESSNESS

**DOI:** 10.1093/geroni/igae098.2790

**Published:** 2024-12-31

**Authors:** Ashley Ritter, Luke Hoban, Rebecca Brown, Meagan Cusack, Helene Moriarty

**Affiliations:** Hunter-Bellevue School of Nursing, Yardley, Pennsylvania, United States; NewCourtland, Philadelphia, Pennsylvania, United States; University of Pennsylvania, Philadelphia, Pennsylvania, United States; University of Pennsylvania, Philadelphia, Pennsylvania, United States; Villanova University, Villanova, Pennsylvania, United States

## Abstract

The number of older adults experiencing homelessness is on the rise nationally, with earlier incidence of geriatric syndromes that jeopardize community living occurring at younger ages in this population. NewCourtland, a provider of housing and health services in Philadelphia, began a program serving older Veterans who experience homelessness with complex health and social needs in 2019. This study examined the feasibility and acceptability of program implementation among program participants and staff. We conducted focus groups with program staff (n=10) and residents (n=9) using a semi-structured interview guide. Transcripts were analyzed using inductive codes derived from the CFIR framework. Key themes regarding program feasibility and acceptability include the attainment of new space and autonomy coupled with the need for goal setting to achieve stability in a community setting. Residents and staff both reported that moving into stable housing was a critical step towards community re-integration for participants. This allowed participants to develop regular habits and boundaries in their personal lives. The program’s focus on goal setting motivated participants to work with the staff members to meet their needs. However, this was only possible if trust had been established on both sides. Clear rules and consistent interaction with staff were essential, both to achieve participant buy-in and reduce staff burnout over time. Findings provide practical insights into how to improve supportive housing programs and enhance community reintegration for older adults with experiences of homelessness and complex health and social needs.

